# Supplementary material for: The Construction and Exploration of a Comprehensive MicroRNA Centered Regulatory Network in Foxtail Millet (Setaria italica L.)
Source: Front Plant Sci. 2022 May 6;13:848474. doi: 10.3389/fpls.2022.848474 (PMC9121102; doi:10.3389/fpls.2022.848474)
Supplement: Supplementary file 4 [file Data_Sheet_4.zip › Sit-miR2275b_Seita.7G310200.1_504_TPlot.pdf]

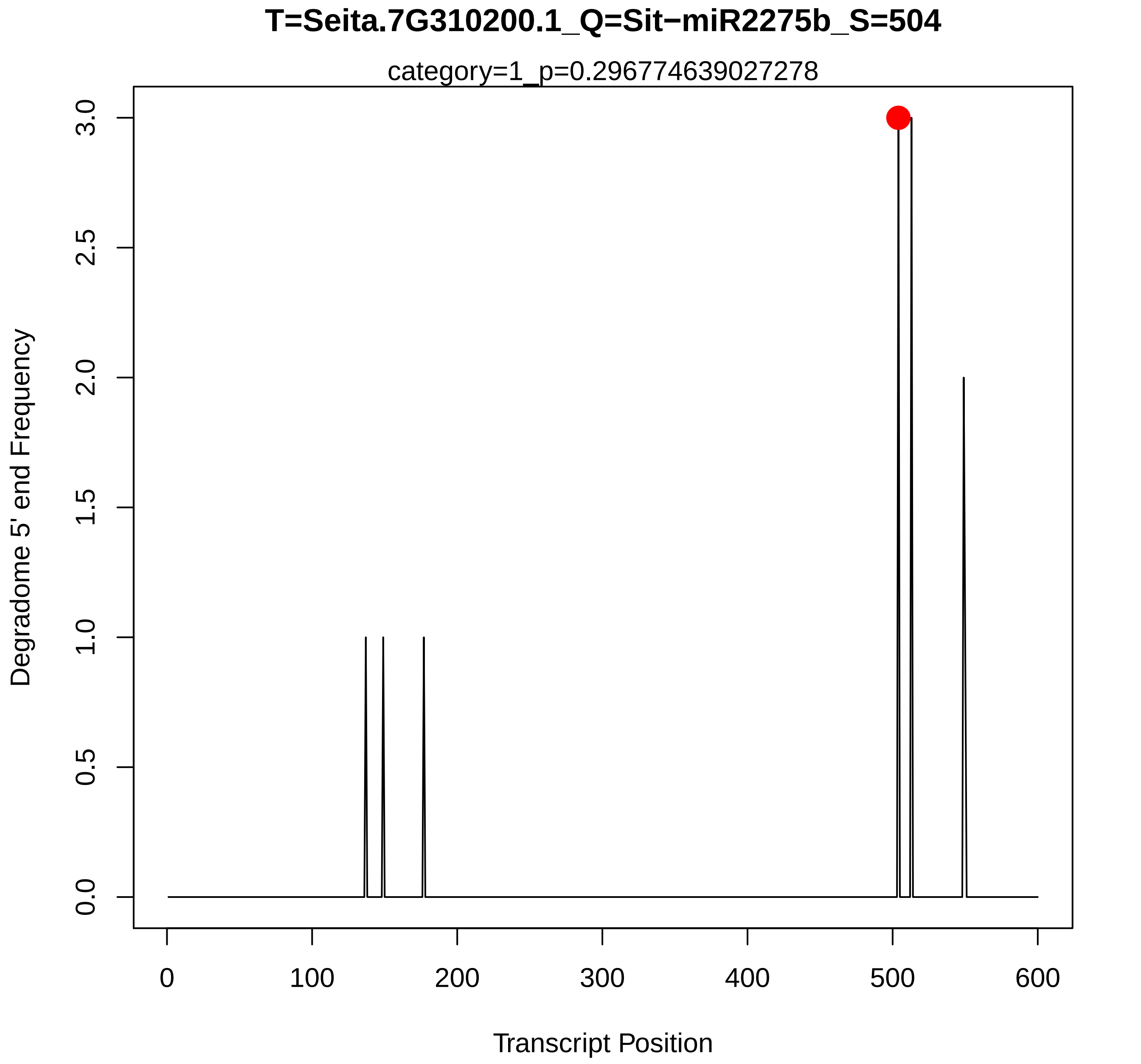
T=Seita.7G310200.1_Q=Sit−miR2275b_S=504
category=1_p=0.296774639027278
Degradome 5' end Frequency
0.0
0.5
1.0
1.5
2.0
2.5
3.0
0
100
200
300
400
500
600
Transcript Position
